# Attitude and preferences towards oral and long-acting injectable antipsychotics in patients with psychosis in KwaZulu-Natal, South Africa

**DOI:** 10.4102/sajpsychiatry.v26i0.1509

**Published:** 2020-07-27

**Authors:** Krishanand R. Roopun, Andrew Tomita, Saeeda Paruk

**Affiliations:** 1 Discipline of Psychiatry, University of KwaZulu-Natal, Durban, South Africa; 2 Centre for Rural Health, University of KwaZulu-Natal, Durban, South Africa; 3 KwaZulu-Natal Research Innovation and Sequencing Platform (KRISP), College of Health Sciences, University of KwaZulu-Natal, Durban, South Africa

**Keywords:** drug attitude, drug preference, antipsychotic, oral and long-acting injectable antipsychotic, South Africa

## Abstract

**Background:**

Patient attitudes to and satisfaction with their treatment are associated with improved adherence. There is a paucity of data on patient drug attitudes and preference to oral compared to long-acting injectable (LAI) antipsychotic treatment.

**Aim:**

To describe patients attitudes and preferences towards oral versus LAI antipsychotic formulations and explore factors associated with their drug attitudes.

**Setting:**

Two psychiatric hospitals in KwaZulu-Natal, South Africa.

**Method:**

A cross–sectional survey of 140 adult outpatients with schizophrenia spectrum disorders receiving LAI with or without oral antipsychotics (a total of 70) were compared to patients receiving oral antipsychotics only (*N* = 70). A sociodemographic-clinical questionnaire, chart review and the Drug Attitude Inventory scale (DAI–30) were used.

**Results:**

Of the 140 participants, 98 (70%) preferred the medication formulation currently prescribed, and 132 (94.3%) reported a positive drug attitude towards their antipsychotic medication. The adjusted regression analysis indicated that study participants who were currently on a formulation that matched their preference scored better on the DAI-30 than individuals with a mismatch in use and preference (*p* < 0.04). In terms of covariates, we found, on one hand, that study participants who are divorced (compared to single) with schizophrenia diagnosis (compared to other psychotic or schizoaffective disorder) are more likely to have lower score on DAI-30. On the other hand, we found that study participants with a higher household income and longer duration of the psychotic illness were associated with greater DAI-30 score.

**Conclusion:**

The majority of participants preferred their current oral and LAI formulation. Drug attitude was influenced by several factors, including matched medication use. Focused psychoeducation should be considered for newly diagnosed, lower socio-economic groups and patients with non-affective psychosis to improve drug attitude.

## Introduction

Schizophrenia is a chronic and disabling illness, with most patients experiencing multiple relapses during their illness; this risk is diminished by maintaining antipsychotic drug treatment.^1^ The two main antipsychotic formulations are oral and long-acting injectables (LAI), the former being prescribed enterally and the latter administered parentally intra-muscularly.^2^ The main indication for LAI antipsychotics is maintenance treatment for people with schizophrenia or schizophrenia spectrum disorders for whom relapse prevention is especially important.^3^ Recently, large-scale naturalistic studies show major benefits of LAI versus oral antipsychotics for this relapse prevention, particularly among patients with first episode psychosis, which can be enhanced by including an assertive monitoring programme.^4,5,6,7^

The National Institute for Health and Care Excellence (NICE) guideline for medication adherence recommends that patients should be as involved as possible in decisions about the choice of medicines that are prescribed for them, and that clinicians should be aware that illness beliefs and beliefs about medicines influence adherence. The NICE guideline for schizophrenia further emphasises the importance of patient choice rather than specifically recommending a class or individual antipsychotic as first-line treatment.^8^

Drug attitude is defined as a settled way of thinking or feeling about something;^9^ questionnaire surveys have been used to measure drug attitude, being regarded as an indirect measure of medication adherence.^10^ Studies assessing drug perceptions have most commonly used the Drug Attitude Inventory (DAI) scale by Hogan to assess the patients’ perception towards medication. The DAI has been designed to assess the subjective drug attitude toward antipsychotic medication of people with schizophrenia, which may affect compliance with treatment.^11^ Nielsen and colleagues suggest that the DAI is a reliable predictor of poor adherence to treatment in schizophrenia.^12^ The DAI has also been modified to a shorter 10 item scale, the DAI-10, which has only 10 of the 30 items of the original scale.^11,13^

Preference is defined as a greater liking for one alternative over another or other options.^9^ A medical definition of preference can be statements made by individuals regarding the relative desirability of a range of health experiences, treatment options or health states.^14^ Drug preference may influence drug attitude and potentially adherence. Drug attitude may also be influenced by several other factors of which severity of side effects is the principal correlate. Other factors, such as younger age, male gender, employment, higher family income, perceived drug efficacy, lower expense, necessity for taking treatment and urban residence, are also associated with improved drug attitude.^15,16^ A study in Nigeria using the DAI-10 found that the attitude of patients with schizophrenia towards medication was significantly influenced by variables such as lack of insight, functional level, severity of illness and side effects.^13^

Perceived well-being, spiritual causation, financial challenges and stigma were also associated with poor adherence to antipsychotic medication.^17^ A study at a South African public sector specialist psychiatric outpatient clinic on a communication intervention with patients showed that the most common response to ‘reasons for non-adherence’ was forgetfulness (28.6%), or that they did not have anybody to remind them to take medication (24.2%). In the same study, 13% of participants reported that the reason for their non-adherence to medication was their perceived reactions to the medication.^18^

Studies from Africa on patient attitude to LAI compared to oral antipsychotic preparations are limited. To the authors’ best knowledge there is no study of patient perspective of LAI antipsychotics compared to oral preparation in Africa. The only study on attitude towards LAI antipsychotics in Nigeria was based on clinician attitude towards depot antipsychotics.^19^ This current study, therefore sought to understand patients’ drug attitude and preference of oral compared to LAI antipsychotic medications.

## Aim

The aim of this study was to describe the attitude and preferences towards oral compared to LAI antipsychotic formulations among patients with a psychotic disorder (schizophrenia spectrum disorders) attending at either one of the two psychiatric hospitals in KwaZulu-Natal (KZN), South Africa.

## Methods

The study objectives were to describe and compare the sociodemographic and clinical profiles of two groups of patients, those taking oral antipsychotic only and those taking LAI and/or oral antipsychotic medication, in relation to their drug attitudes and personal preferences to medication. In addition, associations between sociodemographic and clinical factors and total drug attitude score in patients receiving oral compared to LAI with or without oral antipsychotic preparation are described. The LAI group was subdivided into LAI only and those on combination of LAI and oral antipsychotic in sub-analysis.

### Sample and procedure

A descriptive, cross-sectional study involving patient interviews, chart reviews and questionnaire interviews with adult outpatients receiving either LAI with or without oral antipsychotic, or oral antipsychotic medication only was conducted at two psychiatric hospitals in KZN. The data was collected over a seven-month period, from July 2018 to January 2019. A random sample of patients attending the hospital clinics and meeting inclusion criteria were invited to participate in the study.

### Participants

The study was conducted at the two outpatient departments, at which 140 adult patients (aged 18 to 65 years) were randomly recruited from the outpatient services. Seventy patients were prescribed LAI with or without another oral antipsychotic medication, while 70 were on oral antipsychotic medication only. All participants were on antipsychotics for at least six months, had a diagnosis of a primary psychotic disorder based on the DSM 5 criteria for schizophrenia and schizophrenia spectrum disorders, did not require admission and were able to provide informed consent. The diagnosis was confirmed by clinical interview using the DSM 5 criteria and chart review. Participants were literate, able to speak English and provided written consent.

### Study sites

The two study sites are specialist psychiatric hospital services with inpatient and outpatient services that serve as the referral centres to the general hospitals in the eThekwini (site 1) and uMsunduzi (site 2) municipalities in KZN.

Of the 140 patients, 93 were from site 1 and 47 were from site 2. The first psychiatric hospital has approximately 900 adult outpatients per month and the second hospital has 450 adult outpatients per month.

## Measures

### Sociodemographic questionnaire

The self-designed questionnaire was administered for this study, with data on demographic factors, such as age, gender, educational level, race, marital status and household monthly income being collected. The questionnaire included clinical factors such as primary psychiatric diagnosis (stratified into schizophrenia, schizoaffective disorder and other psychotic disorders), comorbid psychiatric diagnoses (as per DSM 5 criteria and chart review), comorbid medical disorders, number of admissions in past and treatment details, such as medication type and duration based on a review of the literature.

In addition, self-reported personal treatment preference towards oral or LAI formulation was also collated using a structured question with the option of selecting oral only or LAI only as their preferred treatment option. Participants selected their main reason for formulation preference. All interviews and chart reviews to confirm the clinical factors were conducted by the principal investigator. Interviews were conducted in English.

### Drug Attitude Inventory

The DAI is a 30-item self–report inventory that focuses on the subjective effects of neuroleptic medication in patients with schizophrenia to understand the factors that influence drug attitude.^20^ While the DAI has not been validated in SA, it was used in a study in KZN to assess patient drug attitude towards clozapine.^21^

The DAI has been used in many developing countries, such as Ethiopia and Nigeria, as well as in studies outside Africa, including in the Clinical Antipsychotic Trial of Intervention Effectiveness (CATIE).^22^ It has shown high internal consistency in the 30-item scale, with preliminary validation in the form of discriminant classification, and accurately assigned 89% of the sample to compliant and non–compliant groupings.^20^

The DAI has 15 items that are scored as TRUE and 15 as FALSE in the case of a fully compliant patient (positive subjective response) as per the questionnaire. A correct answer to these items is scored as +1, while an incorrect response is scored as –1. The final score is the sum of the total of pluses and minuses. A positive total score suggests a positive subjective response or attitude, while a negative score means a negative subjective response or attitude. The maximum score is 30 if all responses are correct. The DAI score has been reported to predict adherence in schizophrenia,^23,24^ with a negative score predicting non-adherence.^23^

### Pilot testing of measures

The sociodemographic questionnaire and the DAI were pilot tested on 10 patients meeting the same inclusion criteria from the same study sites to determine its ease of understanding and the wording of questions. No problems were reported by patients with understanding the questions and the questionnaire was not modified.

## Statistical analysis

Three analyses were conducted for this investigation to address the study objectives. Firstly, descriptive statistics were used to summarise the participants’ sociodemographic and clinical profiles (including attitude towards medication). Secondly, the sociodemographic and clinical profiles were compared between three groups of participants who were prescribed: (1) oral medication only, (2) LAI only or (3) both, based on chi-square (χ2) test.

Thirdly, multivariate regression models were used to investigate the association between concomitance of medication (i.e. matching of preferences and actual treatment) and the DAI scores using a regression model.

The model was adjusted by sociodemographic (gender, race, age, marital status, education and household income) and clinical covariates (psychiatric diagnosis, history of rehospitalisation due to medication discontinuation, number of past psychiatric admissions and duration of illness). Given the skewness of the DAI score, cubic data transformation was applied to normalise the distribution, which performed better than other transformation (e.g. square, square root, log). A significance of *p* < 0.05 was used for statistical significance testing, and the data were analysed using Stata Version 15.

### Ethical consideration

The study was approved by the University of KwaZulu-Natal’s Biomedical Research Ethics Committee (Ref. No.: BE054/18). Permission was also obtained from the hospitals and the Department of Health.

## Results

### Sociodemographic and clinical profile

Of the 140 participants, the majority were male (*N* = 97, 69.29%) and single (*N* = 97, 69.29%), while the most common primary diagnosis was schizophrenia (*N* = 96, 68.57%). The mean duration of illness of the participants was 11.63 years (standard deviation [SD] 10.3) and median of 8 years (interquartile range [IQR] 16). Table 1 summarises the sociodemographic and clinical data for all the study participants.

**TABLE 1:** Sociodemographic and clinical profiles of participants.

Variable	*N* = 140	%
**Gender**		
Male	97	69.29
Female	43	30.71
**Race**		
Black people	58	41.43
White people	9	6.43
Indian people	51	36.43
Mixed race people	22	15.71
**Age categories**		
19–24	24	17.14
25–34	38	27.14
35–49	44	31.43
50+	34	24.29
**Marital status**		
Never married	97	69.29
Married	25	17.86
Divorced or widowed	18	12.86
**Educational attainment (highest)**		
Grade 11 or lower	67	47.86
Grade 12	31	22.14
Higher than 12	42	30.00
**Household income**		
R2500 or lower	39	27.86
R2501 – R6000	65	46.43
R6000 or higher	36	25.71
**Primary diagnosis**		
Schizophrenia	96	68.57
Schizoaffective disorder	35	25.00
Other	9	6.43
**History of rehospitalisation due to medication discontinuation**		
Yes	73	52.14
No	67	47.86
**Number of admissions**		
≤ 2	74	52.86
3–5	44	31.43
≥ 6	22	15.71
**Medication group**		
LAI only	18	12.86
Oral only	70	50.00
LAI and oral	52	37.14
**Medication preference**		
Oral	96	68.57
LAI	44	31.43

LAI, long-acting injectable antipsychotic treatment.

### Patients’ drug attitude and preference

Of the 140 patients, 70 were on oral medication only, while in the LAI group (*n* = 70, 50.00%), 18 (12.86%) were prescribed LAI formulation only and 52 (37.14%) were on concomitant oral and LAI medication. In addition, of the 140 participants, 96 (68.57%) preferred oral antipsychotics only. Forty-four (31.43%) preferred LAI antipsychotics preparation.

Table 2 summaries the sociodemographic data for participants based on oral only, LAI only, LAI and oral antipsychotic treatment. To highlight some results, the majority of study participants preferred their current medication rather than another formulation (oral only or LAI only) option. As an example, of the 70 on oral antipsychotic only, 62 (88.57%) preferred this formulation. Similarly, of the 18 on LAI only, 11 (61.11%) preferred this formulation (or 36/70 – 51.43% – when both data from the LAI only and LAI and oral groups are combined).

**TABLE 2:** Sociodemographic and clinical profiles of participants stratified by type of antipsychotic medication formulation.

Variable	LAI only (*n* = 18)		Oral only (*n* = 70)		LAI and Oral (*n* = 52)		Total (*N*)	Test statistics
	*n*	%	*n*	%	*n*	%		
**Gender**								χ2(2) = 0.56, *p* = 0.76
Male	12	12.4	47	48.5	38	39.2	97	
Female	6	14.0	23	53.5	14	32.6	43	
**Race**								χ2(6) = 4.78, *p* = 0.57
Black people	7	12.1	33	56.9	18	31	58	
White people	1	11.1	4	44.4	4	44.4	9	
Indian people	5	9.8	23	45.1	23	45.1	51	
Mixed race people	5	22.7	10	45.5	7	31.8	22	
Total	18	12.9	70	50	52	37.1	140	
**Age categories**								χ2(6) = 9.58, *p* = 0.14
15–24	3	12.5	11	45.8	10	41.7	24	
25–34	4	10.5	20	52.6	14	36.8	38	
35–49	3	6.8	28	63.6	13	29.5	44	
50+	8	23.5	11	32.4	15	44.1	34	
**Marital status**								χ2(4) = 2.67, *p* = 0.62
Single	10	10.3	48	49.5	39	40.2	97	
Married	5	20	12	48	8	32	25	
Divorced or widowed	3	16.7	10	55.6	5	27.8	18	
**Educational attainment (highest)**								χ2(4) = 2.42, *p* = 0.66
Grade 11 or lower	10	14.9	34	50.7	23	34.3	67	
12	3	9.7	18	58.1	10	32.3	31	
Higher than 12	5	11.9	18	42.9	19	45.2	42	
**Household income**								χ2(4) = 3.18, *p* = 0.53
R2500 or lower	7	17.9	15	38.5	17	43.6	39	
R2501 – R6000	7	10.8	35	53.8	23	35.4	65	
R6001 or higher	4	11.1	20	55.6	12	33.3	36	
**Diagnosis**								χ2(4) = 2.33, *p* = 0.68
Schizophrenia	12	12.5	48	50	36	37.5	96	
Schizoaffective disorder	6	17.1	16	45.7	13	37.1	35	
Other	0	0	6	66.7	3	33.3	9	
**History of rehospitalisation due to medication discontinuation**								χ2(2) = 1.56, *p* = 0.46
Yes	11	15.1	33	45.2	29	39.7	73	
No	7	10.4	37	55.2	23	34.3	67	
**Number of admissions**								χ2(4) = 1.05, *p* = 0.90
≤ 2	9	12.2	40	54.1	25	33.8	74	
3–5	6	13.6	20	45.5	18	40.9	44	
≥ 6	3	13.6	10	45.5	9	40.9	22	
**Medication preference**								χ2(2) = 27.04, *p* < 0.01
Oral	7	7.3	62	64.6	27	28.1	96	
LAI	11	25.0	8	18.2	25	56.8	44	

LAI, long-acting injectable antipsychotic treatment.

When all groups are combined, 98 (70) of the 140 participants preferred the medication formulation currently prescribed. Next, we investigated the reasons of treatment preference. Of the 44 participants who preferred LAI only (not shown in Table 2), 31 (70.45%) reported they preferred this because there is ‘less medication to take’, followed by ‘symptoms are better’ (*n* = 11, 25.00%). The most common reasons reported for oral medication only preference was ‘afraid of injection’ (*n* = 41, 43.62%), ‘side effects of LAI’ (*n* = 27, 28.72%) and ‘symptoms are better’ (*n* = 26, 27.66%) (not shown in Table 2).

#### Drug attitude inventory score

The mean DAI score was 16.65 (SD 9.53) with a median score of 18 and IQR of 12 for all participants. The majority (*n* = 132, 94.29%) of participants, irrespective of current antipsychotic formulation, reported a positive total DAI score (≥ 0) indicating a positive subjective response or attitude. Excluding 0 score on DAI, 130 (92.86%) study participants had a DAI score greater than 0.

The total number of correct responses to the individual items on the DAI (*n* = 3266, 77.8%) compared favourably to that of incorrect responses (*n* = 934, 22.2%). The most correctly answered question for the LAI and oral groups was question 30 of the DAI (‘By staying on medications I can prevent myself getting sick’).

The most common incorrectly answered questions for the LAI group were question 12 (‘Medication makes me feel tired and sluggish’ – correct response false as per DAI) and question 27 (‘I am given medication to control behaviour that other people [not myself] don’t like’). Question 27 of the DAI was also most commonly incorrectly answered by the participants in the oral antipsychotic only group. Question 20 (‘It is unnatural for my mind and body to be controlled by medication’) of the DAI was incorrectly answered by 52 participants in total, 28 (40.0%) on LAI antipsychotic and 24 (24.3%) participants on the oral antipsychotic.

The analysis for the differences in attitude on the individual DAI statements (the selected 10 that are included in the DAI-10) between the participants in the two groups (oral compared to LAI with or without oral) showed no statistically significant differences between them (analysis not shown).

### Association between sociodemographic and clinical variables and Drug Attitude Inventory score using a regression model

The association between the sociodemographic and clinical variables and the total DAI score using a regression model are summarised in Table 3. The main finding was that those who were currently on a formulation that matched their preference scored better on the DAI than individuals with a mismatch in use and preference (*p* < 0.04) based on adjusted regression models. In terms of covariates, we found, on one hand, that study participants who are divorced (compared to single) with diagnosis with schizophrenia (compared to other psychotic or schizoaffective disorder) are more likely to have a lower score on the DAI. On the other hand, we found that study participants with a higher household income and longer duration of the psychotic illness were associated with a greater DAI score.

**TABLE 3:** Association between medication preference and Drug Attitude Inventory scale score using regression model.

Variable	Adjusted β	Standard error	*t*	*p*
**Gender**				
[Male]	-	-	-	-
Female	−7.90	14.27	−0.55	0.58
**Race**				
[Black people]	-	-	-	-
White people	24.89	27.76	0.90	0.37
Indian people	−7.91	17.01	−0.47	0.64
Mixed race people	−30.50	19.16	−1.59	0.11
**Age categories**				
[19–24]	-	-	-	-
25–34	−7.12	19.22	−0.37	0.71
35–49	10.08	22.71	0.44	0.66
50+	−1.35	28.08	−0.05	0.96
**Marital status**				
[Never married]	-	-	-	-
Married	−17.79	17.90	−0.99	0.32
Divorced or widowed	−**63.86**	20.41	−3.13	< 0.01
**Educational attainment (highest)**				
[Grade 11 or lower]	-	-	-	-
12	−6.22	15.44	−0.40	0.69
Higher than 12	1.27	15.35	0.08	0.93
**Household income**				
[R6000 or higher]	-	-	-	-
R2500 or lower	7.93	17.74	0.45	0.66
R2501 − R6000	37.85	16.48	2.3	0.02
**Diagnosis**				
[Schizoaffective disorder]	-	-	-	-
Schizophrenia	−**38.44**	15.09	−2.55	0.01
Other	−6.83	28.36	−0.24	0.81
**History of rehospitalisation due to medication discontinuation**				
[Yes]	-	-	-	-
No	−3.39	14.09	−0.24	0.81
**Number of admissions**				
[≤ 2]	-	-	-	-
3–5	−3.37	15.84	−0.21	0.83
≥ 6	−22.43	20.14	−1.11	0.27
**Duration of illness**				
In years	1.93	0.92	2.11	0.04
**Medication preference**				
[Divergent]	-	-	-	-
LAI individual preferring LAI	49.61	23.28	2.13	0.04
Oral individual preferring oral	27.35	13.53	2.02	0.04

LAI, long-acting injectable antipsychotic treatment.

Note that adjusted β are transformed. Caution is warranted when interpreting.

## Discussion

The study aimed to describe the drug attitude and preferences towards oral compared to LAI antipsychotics among patients with a psychotic disorder (schizophrenia spectrum disorders as per DSM 5) attending either one of the two psychiatric hospitals in KZN. The DAI was utilised to measure drug attitude and may indirectly predict patient adherence to psychotropic medication.^12,13^ The key findings of this study were that patients generally preferred the current medication formulation that they were prescribed, while the majority preferred oral medication compared to LAI.

### Medication profile

Long-acting injectable formulation is usually viewed as a monotherapy modality to improve medication adherence adherence; however, some evidence still exists that combination with oral antipsychotic formulations does occur, potentially representing a form of polypharmacy.^25,26^ In this study more than half of the patients on LAI medication (*n* = 52 of 70, 74.29%) were on concomitant oral and LAI formulations. This is consistent with another study where the concomitant use of both formulations was 75.9%.^27^ Several rationales for such practice has been reported such as failure of previous taper, treatment resistance, patient preference and in process of slow taper.^28^ Antipsychotic polypharmacy is reported to increase the risk of medication-related adverse events and drug-drug interactions, increase the use of additional medications to manage collateral side effects and decrease adherence with medication due to the complexity of the prescription.^29,30,31^ Monotherapy helps to reduce the complexity of the medication regimen, reducing associated side effects and allowing clinicians to accurately evaluate patient’s response to the antipsychotic.^29,32^ Hence, it may be prudent to review or rationalise polypharmacy prescriptions in patients on LAI antipsychotic medications to decrease side effect burden and improve adherence.

### Patients’ drug attitudes using the Drug Attitude Inventory and personal preferences towards oral versus long-acting injectable antipsychotic formulations

Patients’ preferences towards antipsychotic medication formulations in general – whether LAIs or oral formulations – are associated with several factors, including current formulation, symptom remission, functioning, side effects and treatment outcomes.^15,33^ In this study, the majority of patients preferred their current antipsychotic formulation, which is consistent with reports that patients tend to favour their current antipsychotic formulation when asked to state a preference.^23,34,35^ In this study, the participants’ report of main reason for preference of the formulation was having a lower pill load (for LAI) and symptom relief in both LAI and oral groups. This is consistent with the literature that patients’ perceived subjective well-being and treatment response may influence medication preference and attitude.^36^ Thus the assessment of subjective well-being is helpful for selecting the optimal drug for the individual patient.^37^ This reinforces the need to involve the patient in decision-making regarding their care. The findings in this small sample, however, need to be treated with caution and will need to be further explored in a larger qualitative study.

In this study, most participants reported a positive drug attitude score, which is consistent with the literature.^13,15,38,39^ This may also be due to sample bias, as participants were attendees at psychiatric hospitals, and thus treatment defaulters in the community were not surveyed, which is supported by findings in other studies. This suggests that such a sample often excludes patients who are non-adherent and likely to have more negative views about their medications.^40,41^ Another reason for the high DAI scores among participants in this study may be subjective responses to the DAI by participants. A positive DAI score may be also considered as a predictor of adherence,^12,13^ with a systematic review that analysed the relationship between medication adherence and drug attitude toward medication, demonstrating a significant positive association between them.^42^

### Associations between sociodemographic and clinical factors and total drug attitude score

This current study showed an association between negative drug attitude and divorced or separated status, suggesting that individuals with a negative subjective response or attitude are more at risk of non-adherence.

While there were no studies assessing marital status and drug attitude, studies assessing the association of marital status and medication adherence (predicted by the DAI)^12,13^ have yielded mixed results. Studies have reported no association^38^ to being married being associated with improved adherence, and being separated or widowed being associated with non-adherence.^39^ Various publications indicate that being single usually leads to a greater preponderance of non–adherence, which can be secondary to less psychological and financial support, in addition to having less help to bring them to the patient’s appointment.^43^ This may be explained by divorced or separated individuals having less social support, with another study in South Africa showing that non-adherence may be due to there being nobody to remind patients to take their medications.^18^ This suggests the need for wider involvement of the family in treatment management.

The literature reports that financial burden and unemployment were associated with medication non-adherence in patients with schizophrenia,^44^ and non-adherent groups of patients had a lower monthly household income per capita compared to the adherent patients.^45^ The current study found that those who had a higher income had a more positive attitude towards their medication, which is consistent with the literature.^13,44,45^ This suggests the need to focus psychoeducation on these vulnerable groups and to address social adversities to improve patient outcomes.

More positive drug attitudes have been consistently found to be associated with the ‘insight’ into the presence of a mental disorder.^46,47,48,49^ Patients affected with schizophrenia have been shown to have poorer insight into their illness when compared to those with schizoaffective disorders.^50^ Those with schizophrenia also display poorer insight into having a mental disorder and on the social consequences thereof compared to patients with bipolar disorder.^49,51^ Consistent with these previous findings, this study also found that those who had a diagnosis of schizophrenia were more likely to score more negatively on the total drug attitude score towards their medication than those with a diagnosis of schizoaffective disorder and other psychotic disorders.

This finding highlights the need to intensify psychoeducation efforts for those diagnosed with schizophrenia to potentially improve adherence.

The association between duration of illness and drug attitude score towards psychotropic medication has been established, and shows that the longer the duration of an illness the more favourable the subjective attitude to medication.^38^ In the case of LAI antipsychotic, it was found that acceptance of such a formulation increases with personal experience.^52^ As the psychotic illness progresses, patients tend to gain insight into the condition, increasingly recognising the severity of their problems, and accepting that antipsychotics are a possible solution in reducing those symptoms, which results in a more positive attitude towards the antipsychotic treatment.^53^ It has been shown that psychoeducation for patients with a mean disease duration of 2.57 years was associated with an increase in illness knowledge, and significantly correlated with a better drug attitude towards medication.^54^ Our study’s finding of an association between longer duration of illness and improved drug attitude score towards medication is, therefore, consistent with the literature, and suggests the need to reinforce psychoeducation and focus it more intensively in those initiating treatment for the first time or early in their illness.

The current study failed to find an association between drug attitude score and history of rehospitalisation due to treatment discontinuation or number of admissions. This is not consistent with the literature,^47,55^ as previous studies suggest that patients with a poorer drug attitude score are at increased risk of poorer drug adherence and hence poorer DAI scores were associated with increased relapse and readmission. The lack of association in this study may be due to biased reporting by participants, thus inflating DAI scores, small sample size or sample selection bias as participants were all on treatment, currently adherent and hospitalised in the past six months.

### Limitations

Several limitations have been identified in this study, including that it was urban and hospital-based, which may limit the findings generalisations, although both psychiatric hospitals receive patients from a large referral area that includes rural residents. There may be also patient bias in a hospital-based samples as these patients are often more difficult to treat, hence are prone to relapse, compared to patients referred back to local clinics for further care. The lack of validation of the DAI tool in the local setting and recruiting only English-speaking participants in the study limits the findings further. The sample size may additionally limit the assessment for associations. The cross-sectional nature of the study limits the findings, which may be better assessed longitudinally, as patients surveyed were adherent and attending clinic at the time of survey. There may be under-reporting of non-adherence of medication, as the study relied on subjective reports. In our setting, patients on LAI antipsychotic only were limited in number, as many of those who were assessed as using LAI, were also on oral antipsychotic medication; however, this possibly provides a more realistic picture of current local practice.

Furthermore, this study is limited as insight and side effects were not measured and hence, their effect on DAI was not assessed. Finally, it will be useful to have included collateral information from the caregiver to get more objective data on patients’ drug attitude.

## Conclusion

The key findings of the study are that most participants reported a positive drug attitude towards their current antipsychotic formulations. The majority of participants preferred oral compared to LAI formulation. There was a positive association between drug attitude score and subjective formulation preference, and participants who were on a formulation that matched their preference scored better on the DAI compared to individuals with mismatch in use. The findings suggest the need for considering drug formulation preference and more focused psychoeducation in groups vulnerable to poor drug attitude, such as the newly diagnosed, unemployed, single, those with schizophrenia and those on regimens with polypharmacy.

Further research is required to better assess patient drug attitude in larger longitudinal studies, and to consider interventions to improve drug attitude and possibly adherence.
